# Thorough Specification of the Neurophysiologic Processes Underlying Behavior and of Their Manifestation in EEG – Demonstration with the Go/No-Go Task

**DOI:** 10.3389/fnhum.2013.00305

**Published:** 2013-06-24

**Authors:** Goded Shahaf, Hillel Pratt

**Affiliations:** ^1^Neurology Department, Rambam Health Care Campus, Haifa, Israel; ^2^Evoked Potentials Laboratory, Technion Israel Institute of Technology, Haifa, Israel

**Keywords:** neurophysiologic processes, representation, modeling, analysis, EEG/ERP, go/no-go, ADHD

## Abstract

In this work we demonstrate the principles of a systematic modeling approach of the neurophysiologic processes underlying a behavioral function. The modeling is based upon a flexible simulation tool, which enables parametric specification of the underlying neurophysiologic characteristics. While the impact of selecting specific parameters is of interest, in this work we focus on the insights, which emerge from rather accepted assumptions regarding neuronal representation. We show that harnessing of even such simple assumptions enables the derivation of significant insights regarding the nature of the neurophysiologic processes underlying behavior. We demonstrate our approach in some detail by modeling the behavioral go/no-go task. We further demonstrate the practical significance of this simplified modeling approach in interpreting experimental data – the manifestation of these processes in the EEG and ERP literature of normal and abnormal (ADHD) function, as well as with comprehensive relevant ERP data analysis. In-fact we show that from the model-based spatiotemporal segregation of the processes, it is possible to derive simple and yet effective and theory-based EEG markers differentiating normal and ADHD subjects. We summarize by claiming that the neurophysiologic processes modeled for the go/no-go task are part of a limited set of neurophysiologic processes which underlie, in a variety of combinations, any behavioral function with measurable operational definition. Such neurophysiologic processes could be sampled directly from EEG on the basis of model-based spatiotemporal segregation.

## Introduction

The manner by which the brain represents information is the subject matter of many models (see, for example Markram, 2006). These models often differ significantly in the derived characterization of elementary units of representation and of their inter-relations. Nevertheless, despite significant differences between models, it seems that specific neuronal networks are often considered as the elementary representation units – for instance cortical columns (see, for example Mountcastle, 1997).

The activity of such networks and their inter-relations with other networks is constrained by neuronal infrastructure as well as by anatomical division to functional brain regions. The infrastructure constraints restrict the possible activity pattern of the networks including their overall activity duration. They furthermore restrict the inter-network patterns of effect. The anatomical constraints divide the brain to functional regions. Neuronal networks in each region are believed to represent related entities, in terms of perceptual, motor, or other features (see, for example Mesulam, 1998). Such anatomical constraints may restrict the types of possible representations. Furthermore, they seem to restrict the possible inter-connections between the types of elementary representations due to limitation of anatomical connections between the functional regions (see, for example Mesulam, 1998).

The precise details of both infrastructure and anatomical constraints are still under research. At the infrastructure level, different theories suggest different types of neural code ranging from population rate coding, which considers the network as one unit, to precise spatiotemporal coding, in which the precise temporal timing of spikes from specific neurons could differentiate between representations (see review in deCharms and Zador, 2000). The neural code employed determines the subtlety and versatility of effect of a given network upon other networks, which receive input from it. This effect is also dependent upon the characteristics of input activity summation between its various input networks, affecting a target network (for example, see the suggested role of dendritic tree computation in London and Hausser, 2005). At the anatomical level there are also diverse opinions regarding the precise division to functional regions and sub-regions and regarding inter-region functional connectivity.

Nevertheless, despite variability with regard to detailed characteristics, it seems that significant basic characteristics are shared by many of the major models involving both infrastructure and functional anatomical division to regions. As was stated the majority of models are based upon elementary units of representation in the form of neuronal networks and these units are divided into functional regions, whose inter-connectivity limits the plausible inter-unit interactions. While the precise division to the finer regions is variable between models, there is rather strong evidence concerning the basic functionality of many of the brain regions, and even, although to a lesser extent, regarding inter-regional functional connectivity. Furthermore, despite variability in terms of the activation pattern of the elementary neuronal network, the majority of models restrict the duration of this activation to well below the second timescale (ample data exists regarding typical burst duration of neurons of cortical columns ever since the work of Hubel and Wiesel, 1959). Importantly this timescale of activity is often also well below the timescale of most of behavioral function.

On the basis of these basic similarities we have built a simulation tool, which enables the neurophysiologic modeling of behavioral functions. The simulator is comprised of modular specification of brain regions, and each region is comprised of modular neuronal networks, which are its elementary units of representation. It enables parametric selection of most of the infrastructure and anatomical constraints, so as to support the evaluation of various theories.

We believe it may be of much interest to evaluate the effects of selecting different characteristics for the various parameters in the simulator. However the purpose of this work is to state that, even regardless of the precise characteristics, the mere division of the brain into functional regions, in which activity is implemented in elementary neuronal networks, seems to yield major insights regarding the neurophysiologic processes underlying behavioral function. For this aim we present in a step-by-step manner the challenges we encountered, and possible solutions we reached, while modeling a behavioral task in this neurophysiologic simulator. We selected the go/no-go task as a representative and known example but, as is emphasized throughout the text, this is just an example of the plausibility of utilizing a similar basic approach for modeling any behavioral function and dysfunction, as long as it is defined operationally.

This strict modeling process leads to the derivation of three neurophysiologic processes, which underlie the go/no-go task, each process with its estimated spatial and temporal characteristics. The experimental implementation of the ability to specify the underlying neurophysiologic processes, even with partial spatiotemporal precision, is then demonstrated with EEG/ERP data. First we discuss in general terms how the neurophysiologic processes in the model are expected to manifest in EEG. Next we interpret the ERP literature regarding the go/no-go task in subjects without or with Attention Deficit Hyperactivity Disorder (ADHD). Then we demonstrate that the ERP manifestations of the modeled processes seem to emerge from a comprehensive bottom-up signal analysis. Finally, we demonstrate how effective EEG markers for behavioral function vs. dysfunction (in this case ADHD), could be derived from harnessing differences in spatiotemporal characteristics of the modeled processes to untangle the superposition of the manifestations of the various processes in the sampled EEG signal.

We finish by emphasizing that the modeling of the go/no-go task and the EEG/ERP interpretation and untangling presented, in the context of ADHD, should be considered a mere example of a general approach, applicable to any operationally definable behavioral function or dysfunction. We touch upon the significance of this possibility.

## Thorough Modeling Demonstrated with the Go/No-Go Task

### Representing temporary go/no-go stimulus-response relations

The first challenge we encountered when trying to model the go/no-go process is how to present the temporary relations between the go stimulus and its related response and between the no-go stimulus and its avoid response. Depending on the sensory modality employed, we expect the involvement of certain sensory regions in the go/no-go task: primary sensory, higher unimodal, and heteromodal. Similarly, depending on the specification of the go and avoid responses, we expect the involvement of specific motor-related regions: primary as well as higher motor and prefrontal regions. Specifically, the no-go or avoid response may involve mainly such higher regions (Bruin and Wijers, 2002).

**Figure 1:**
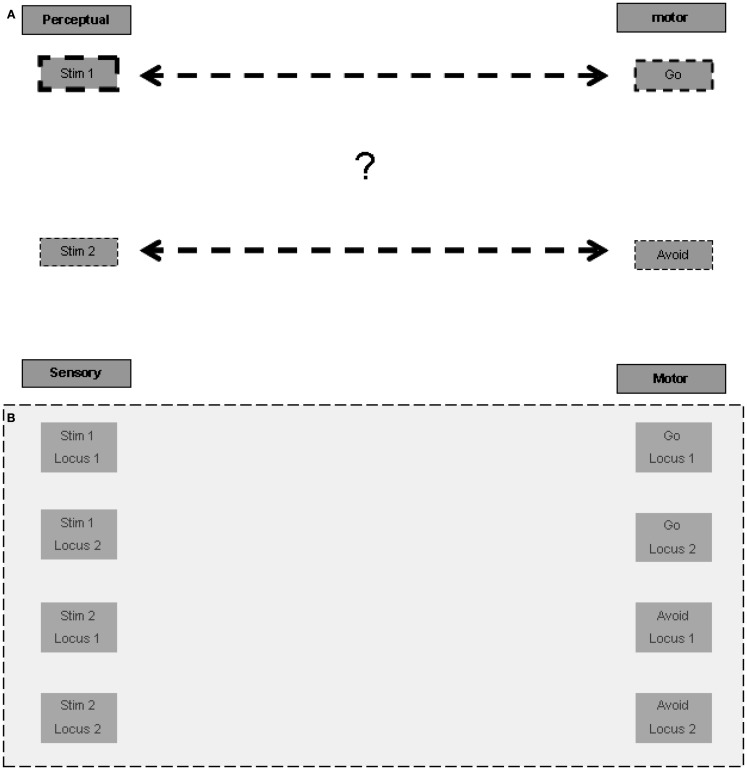

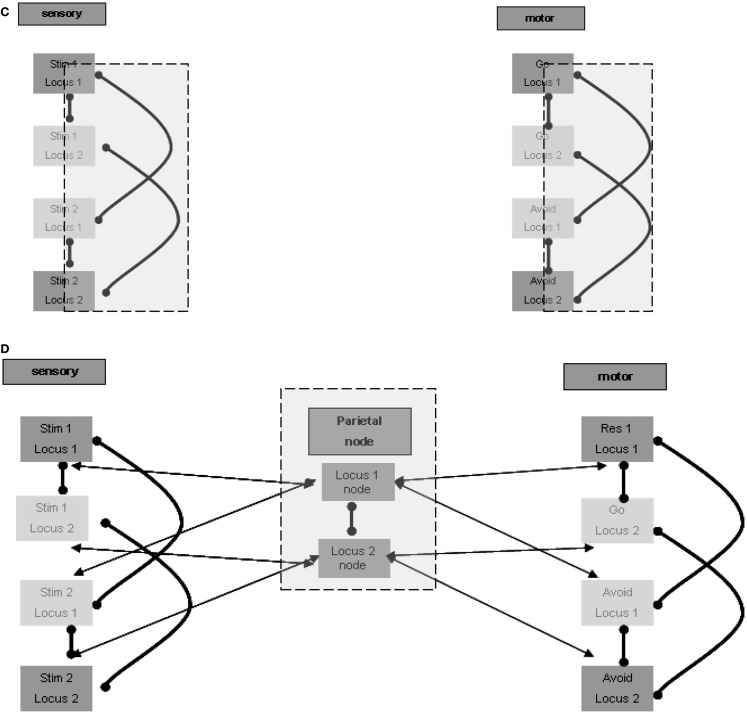
**Representation of temporary stimulus-response relations**. **(A)** The riddle: how are temporary associations formed? **(B)** Spatial multiplication of each stimulus and response (in each of the figures presenting the expanding model, the additive model blocks and connections are emphasized with a gray square). **(C)** Lateral inhibition for selection of one representation in one locus. **(D)** Inter-modality connection according to localization.

The association of a specific stimulus with the go response and of another with the avoid response is arbitrary. The association is not a stable one that may be important to retain permanently in long-term memory in order to promote a specific response to a specific stimulus in other contexts and in the case of other tasks. Therefore, the first question we address is how temporary arbitrary relations are formed for stimulus-response pairs represented in different anatomical regions? This question is illustrated graphically in Figure 1A.

#### Representation of inter-modality relations by “spatial ordering”

A possible answer may be based on allocating a different spatial ordering to each stimulus in the relevant sensory regions, and to each response in the relevant motor regions. Given that the association of stimulus-response pairs is arbitrary, in principle, each stimulus and each response may present in any of the possible ordering locations, and therefore a representation unit should exist for each possible stimulus in each location in the relevant sensory regions. The same holds for each response in the relevant motor regions (Figure 1B). Note that the current task involves only two pairs of stimuli and responses, but other tasks may be based on a larger ordering of loci. There is evidence that multiple representations of the same stimulus take place in different spatial perceptual fields (the spatial cortical mapping of the same stimulus is well established for various sensory modalities), which may be used for such spatially ordered representations.

#### Lateral inhibition within location for selection

The arbitrary and temporary allocation of a specific representation to a specific locus may be based on lateral inhibition between the various representing units relating to the same locus. We do not model the learning of the stimulus-response pairs when the task is first introduced to the subject, and limit ourselves to modeling the following performance of the task. For the sake of simplicity, it may be possible to envision that the first stimulus presented in the learning phase activates the unit representation of the first stimulus at the first locus, laterally inhibiting the activation of unit representations of other stimuli at the first locus. Similarly, the second stimulus presented in the learning phase may activate the representing unit of the second stimulus at the second locus. This representing unit, as opposed to that of the second stimulus at the first locus, is not already laterally inhibited by the representation of the first stimulus at the first locus. It is not unlikely that more elaborate modes of allocation take place, for example such that relate to the significance, intensity, or familiarity of the stimulus. But this elaboration is not essential to our elementary modeling of the go/no-go task.

At this point we wish to speculate another possible type of functional lateral inhibition, between units representing the same stimulus at different loci, assisting in the unique localization of the representation of each stimulus. We are not aware of sufficient supportive evidence for the existence of such more distant lateral inhibition, but some indirect evidence of an enabling mechanism has been reported (hints of this possibility appear, for example, in Grinvald et al., 1994). Although our modeling does not rely upon the existence of such distant lateral inhibition relations, we discuss below the possible implication of their existence.

Similarly, local lateral inhibition relations may also exist in the relevant motor regions between response representations such as go and avoid. Hypothetically, the distant functional inhibition, as suggested above for the sensory regions, may also exist in the relevant motor regions. Figure 1C presents the plausible lateral inhibition relations between the units described. Thus associated stimuli and responses could be allocated to the same spatial ordering loci.

#### Node for activation of spatial ordering

To activate a distant association, for example between the relevant stimulus and the go response, cross-modality excitation by loci must be made possible. In principle such an association can be made directly between all units in any locus in one sensory modality and all units in the same locus in another sensory modality, or in our case, in the motor regions. Such all-to-all locus connectivity is required because, as mentioned above, the relation of a given stimulus to a given response can be arbitrary and temporary. But there is a possible alternative connectivity pattern, which may be more parsimonious and accord better with functional anatomical knowledge. This alternative pattern seems to require a spatial node that maintains reciprocal relations with the relevant localizations in the various modalities. Units in this node may represent such abstract localizations, with possible differentiation between representations of the same locus, based on associations with different subsets of modalities. For example, the spatial node may hold a representation of the first locus associated with first locus representations in the auditory and motor regions, another representation of the first locus associated with auditory and visual regions, etc. It is not unlikely that a super-region providing such functionality is located in the parietal lobes, which are known to participate in spatial perception. The parietal lobes are also considered to be a central player in working memory, and it may be that their role in working memory function relates to such spatial ordering association between relevant modalities. Figure 1D presents the possible reciprocal excitatory connections between the parietal node units and related modality units. Note also the suggested lateral inhibitory relation between the spatial representation units, which reduces the risk of erratic association between different spatial loci in the different sensory and motor modalities. Note further that the spatially local lateral inhibition discussed above in the sensory and motor regions should override the global excitation of all units of a certain locus in the spatial node. Otherwise specific representations at specific loci would not be sound because, as described above, specificity is based on lateral inhibition. It follows that the excitatory effect of the spatial center is weaker than the local lateral inhibition within each region.

To summarize, we suggest that the elementary representation of association between stimuli and responses is based on spatial ordering. From this basic construct it is possible to derive the neuropsychological processes that participate in its maintenance and utilization by relevant stimuli.

### First process: Maintaining the temporary relations

The next challenge we encountered in the modeling process was to maintain these temporary relations over the task time. As was stated in the introduction, there is significant variability in modeling the activation pattern of the neuronal networks that form the elementary units of representation. Nevertheless, that the majority of models restrict the duration of this activation to well below the second timescale. This timescale of activity is often also well below the timescale of many behavioral functions, including that of the go/no-go task.

Therefore, the spatial representation of the temporary stimulus-response relations discussed above is a relatively short process with rapid decay, on the scale of seconds or less, but the behavioral function modeled lasts significantly longer. Maintenance of the representation for longer durations requires either stable reverberation among the above-mentioned centers or the involvement of an external source that interacts with these centers to maintain their activity. The lateral inhibition described above can maintain the initial spatial allocation of representation as long as the spatially allocated representations remain active.

#### Longer maintenance is based on a global reverberation center

The need for an external source to support the reverberation may be derived from the above discussion. Long duration of effective reverberation between the sensory, spatial, and motor regions discussed above requires strong and stable inter-unit connections. Because such strong constraints lack specificity, as discussed above, they may override the intra-modality lateral inhibition, which is essential for the maintenance of distant association, in our case between stimuli and responses.

**Figure 2:**
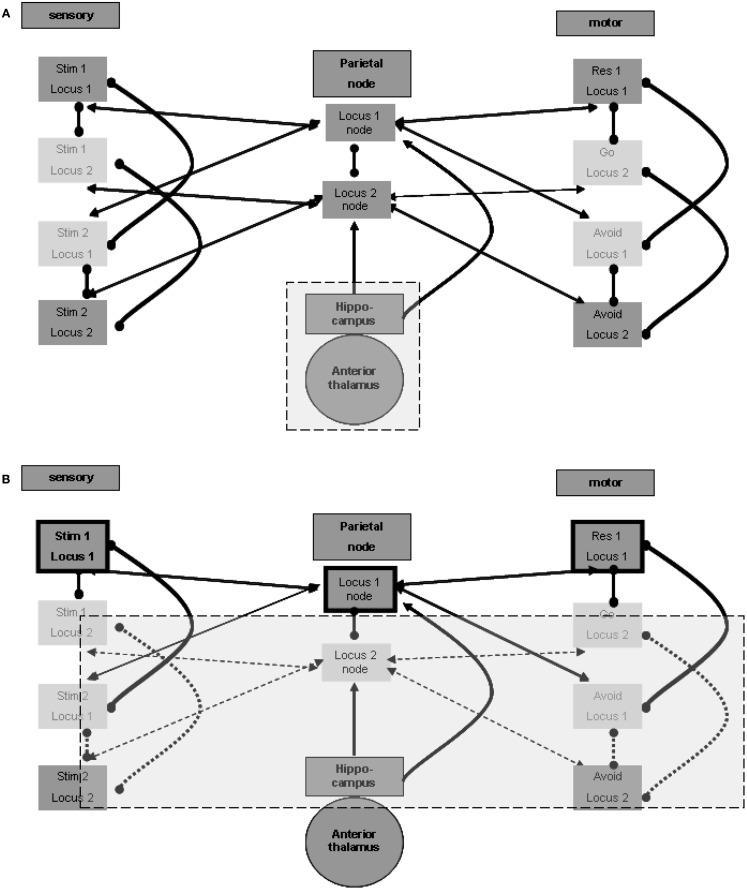
**Memory maintenance process**. **(A)** Possible hippocampal contribution for the maintenance of relations. **(B)** One locus representation without inhibition of the other representation.

The question is which external source promotes the maintenance of activity between relevant regions for a long duration? Although such maintenance has some working memory characteristics, it outlasts the standard time frame of few tens of seconds, usually described for working memory reverberation. The longer duration of maintenance in memory is often related to the hippocampus. Indeed, without going into a detailed description at the level of the neuronal network, there seems to be ample evidence of reverberating oscillations originating in the hippocampal formation. These oscillations appear to promote the activation of relevant cortical regions (see, for example, Sirota et al., 2008), which in our case may be the spatial nodes in the parietal lobe. Figure 2A illustrates this relation.

Three points must be noted in this regard. First, it is likely that the reverberation maintenance involves reciprocal connections between the spatial units at the spatial center region and their relevant hippocampal counterparts, with the hippocampal units acting as enhancers of the reverberation over time. Second, the hippocampal units may also enhance directly the activation of relevant units in the sensory and motor regions at the relevant loci, and different hippocampal units may associate with different modality sets in a manner previously described for the spatial parietal node. Third, there is evidence of especially strong relations between the hippocampal formation and the prefrontal cortex (see, for example, Siapas et al., 2005). Such relations may promote long-lasting activation of working memory, which can then invoke activity of the parietal spatial centers and of the relevant units in the different modalities. We did not include any of these three possible connections in the figures in order to emphasize the minimal working model we can devise. Note, however, that our current limited model probably makes very restricted use of the full span of hippocampal features.

#### Involvement of subcortical loops

The hippocampal-cortical connections also involve significant indirect pathways, which include subcortical regions. The mention of subcortical involvement is of importance because of its potential effect on sampled EEG activity, discussed below. To this end we place special emphasis on thalamic regions and thalamocortical connections. The anterior thalamic nucleus is particularly significant in the context of hippocampal-subcortical-cortical connections (see, for example, Irle and Markowitsch, 1982). We therefore included this region in Figure 2A.

#### Lateral inhibition of representation across loci for simple maintenance

We speculated above about the possibility of distant functional lateral inhibition between presentations of the same stimulus at different loci. Such inhibition enables maintaining the activity of only one locus representation pair of stimulus and response without losing the other association. If, for example, the first stimulus and go response representations selected at the first locus are active, the second stimulus and avoid response representations at the second locus are not inhibited; they can be simply inactive. This is in contrast to the representations of the second stimulus and the avoid response in the first locus, which are locally inhibited, and to the representations of the first stimulus and of the go response in the second locus, which are presumed to be inhibited from a distance. This condition, which can be broadened to more than two representation sets, is shown in Figure 2B, which emphasizes the active representation units and their inter-relations. Lateral inhibition between the two spatial nodes, as presented in the figure, supports the possibility of such simple maintenance. Note that this economic reverberation may affect other neurophysiologic processes. For example, it is possible that if one stimulus (in our case this is often a go-related stimulus) is more frequent, the reverberating set comprises both sensory and motor representations associated with it. This may have an activity-dependent effect of habituation on the evoked response to such a frequent or stronger stimulus, compared with the response to the other stimulus (in our case, the no-go/avoid-related stimulus). Findings reported for the go/no-go task (Nieuwenhuis and Yeung, 2003) seem to support this possibility. Without the distant functional lateral inhibition, both spatial centers and their related associations are required to maintain activity constantly.

### Second process: Evoking the relevant perception by a stimulus

On the basis of the above our next challenge was to model the manner by which stimuli manage to take hold over the maintenance processing, so as to evoke relevant responses.

#### Differentiation between primary and higher unimodal regions

**Figure 3:**
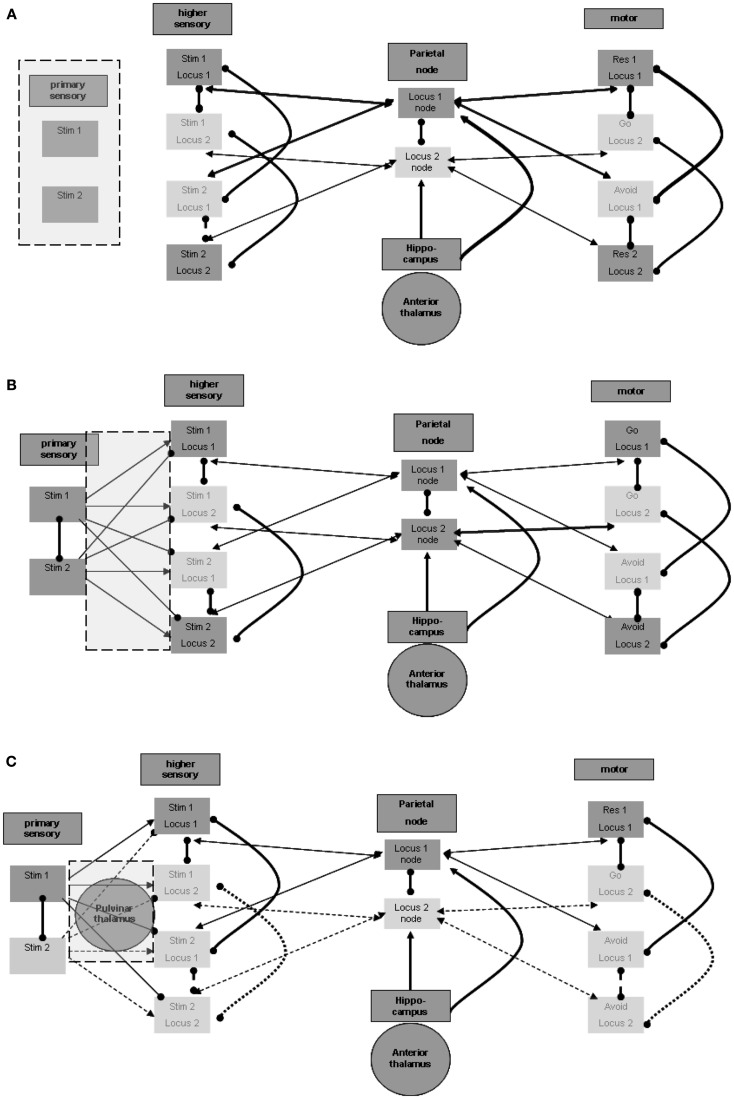
**Perception process**. **(A)** Distinction between primary and higher sensory regions and units. **(B)** Excitation and inhibition from primary to higher sensory regions. **(C)** Possible thalamic involvement in perception.

It appears that there should be a distinction between the units that participate in the maintenance in memory of the stimulus-response associations and the units that represent the sensation of actual stimuli. Otherwise, the brain would have no way of distinguishing between sensed stimuli and maintained memories. Figure 3A illustrates this distinction. The distinction between regions for the purpose of separation between sensed and maintained representations can accord and overlap with the distinction between lower and higher sensory regions, which often relates to levels of processing (for related views in the literature see, for example, D’Esposito, 2007).

#### Forward inhibition from the primary modality region

Maintenance involves associations of representations of stimuli and responses. As shown above, the different stimulus representations in the higher sensory region are maintained actively during sensation and activation of the relevant representation in the primary sensory region. This, however, poses the following difficulty: consider that at a certain time the representation of the first stimulus is activated by sensation in the primary sensory region, and at the same time the representation of the second stimulus is actively maintained in the higher sensory region. This situation, which is not infrequent, may lead to the invocation of the second stimulus – avoid response association, while the first stimulus is being sensed. To prevent this situation, the representation of the second stimulus in the higher sensory region should be temporarily inhibited while the first stimulus is active in the primary sensory region. This inactivation is shown in Figure 3B. In support of this possibility, note evidence of forward inhibition between primary and higher sensory regions, at least for the visual modality in animals. This forward inhibition is similar in extent to lateral inhibition within each region and appears to be significantly greater than, for example, in feedback relations (see, for example, Shao and Burkhalter, 1996). Although only about 10% of such primary-to-higher-region connections seem to be inhibitory, this is also the ratio stated for intra-regional lateral inhibition. It appears that the effect of the inhibitory activations may be stronger because of the activation of tightly coupled local inhibitory networks, possibly by electrical connectivity (see, for example, Galarreta and Hestrin, 2001). Note that inhibitory relations between alternative stimulus representations in the primary sensory region may further assist in reducing wrong activations. Lateral inhibition of this type is also presented in Figure 3B.

#### Thalamic (pulvinar nuclei?) enhancement of perception

As noted above, we want to address possible involvement of subcortical regions, particularly of thalamic nuclei, in the described neurophysiologic processes. This stems from the motivation to relate the modeled neurophysiologic processes with manifestations in the EEG signal, as explained below. The involvement of specific thalamic nuclei in the activation of primary sensory regions is well known. Nevertheless, we wish to mention potential thalamic involvement even in evoking activity in higher unimodal and heteromodal regions. Theory and experimental evidence suggest that posterior thalamic nuclei, including the pulvinar, are involved in activating such cortical regions. This presumably cortico-thalamo-cortical flow via the pulvinar nuclei is reported to be associated with stimuli requiring “perceptual attention.” Although results relating the pulvinar nuclei to visual perception are more prevalent, data on any other sensory modality as well as motor-related data are also present with regard to pulvinar (see, for example, Grieve et al., 2000; Pandya, 1995 in the context of different sensory modalities) or to neighboring posterior thalamic nuclei. Figure 3C incorporates the pulvinar nuclei in the processing between primary and higher sensory regions and emphasizes the active representation units during the perception of the first stimulus. As noted in reference to the spatial parietal node and to the hippocampal formation, different representation units or nuclei in the posterior thalamus may associate with different higher unimodal and heteromodal regions.

#### Overriding activation of relevant spatial node

Evoking the appropriate perception by the stimulus should promote the appropriate response. We suggested earlier that the association of stimuli to responses is likely to be based on a spatial node. But as suggested, such a spatial node is also activated repeatedly by the hippocampus as part of the memory maintenance process. Thus, in principle it is possible that simultaneously with the perception of one stimulus, the other spatial node, and thereby also the other response, are activated as part of the memory maintenance process. This leads to an inappropriate response to the stimulus. To avoid this, the activation of the appropriate spatial node by the representation of the stimulus perception must be sufficiently strong to overcome the lateral inhibition by the other spatial center. After it is activated, the appropriate spatial node inhibits the inappropriate one.

### Third process: Enabling response

Finally we model the manner by which only external stimuli, and not on-going maintenance, evokes response. After a stimulus is sensed, the appropriate response should be enabled. This is in contrast to the internal maintenance discussed above, during which an actual response is not desirable. This distinction seems to require activation by the stimulus of a response-promotion process. The possible implementation of such a process is discussed below.

#### Differentiation between primary and higher motor regions

**Figure 4:**
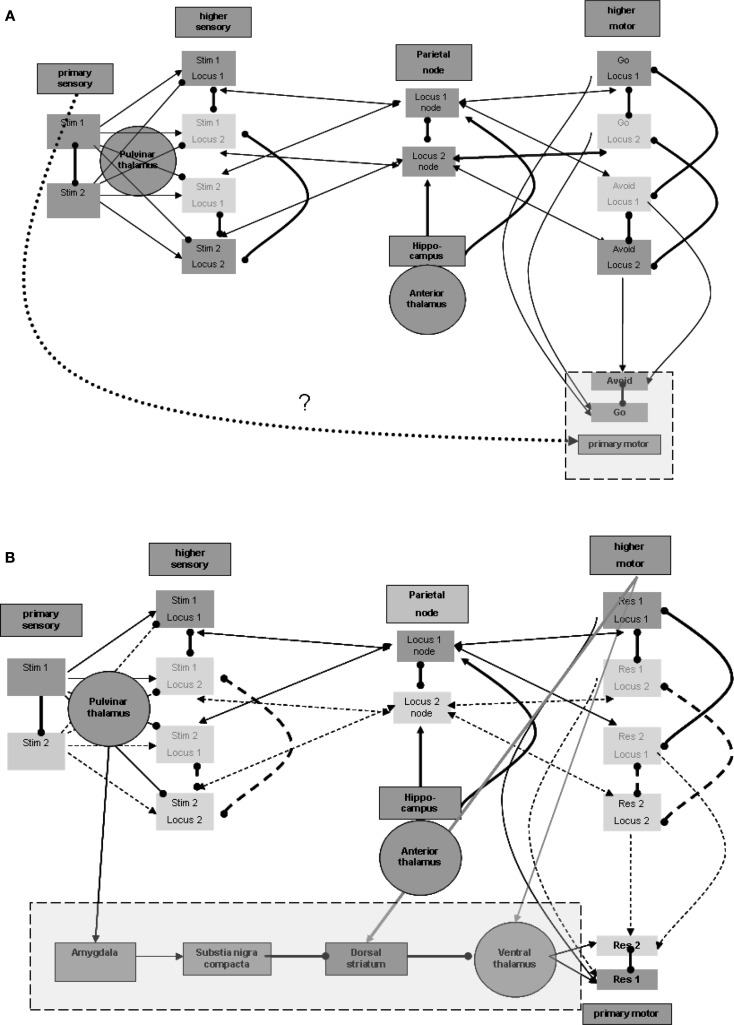
**Responsiveness process**. **(A)** Distinction between higher and primary motor regions. **(B)** Plausible regions participating in promoting responsiveness. Note that connection strengths are depicted as follows: solid line (—) a connection that can take effect on target by itself; dashed line (^a^) – a connection that requires additional connection to take effect upon the target; bold line (**—**) – a connection with overriding effect over regular connections (see the rationale in the text).

Similar to the separation between primary and higher sensory regions, there is also a need to distinguish the primary from the higher motor regions. Without such separation, activations of the motor region during maintenance would invoke the responses represented in it. Furthermore, for the same purpose of disabling motor manifestations during maintenance, input from the higher motor regions should not by itself suffice to activate the representation units in the primary motor region. This condition is illustrated in Figure 4A. An additional process is needed to promote the activation of representations in the primary motor region for the production of actions. This process should be invoked after sensation of the appropriate stimulus. Invoking should not involve higher sensory regions that participate in the maintenance memory, again for the sake of avoiding motor manifestations during maintenance.

#### Promotion of responsiveness without involvement of higher sensation

Thus, the process enabling response should be invoked by the activation of stimulus representation in the primary sensory region, but not by stimulus representations in higher sensory regions. Note again that the pulvinar and posterior thalamic nuclei, which were related above to the activation of higher sensory regions, may receive their driving input from primary sensory regions or even from the precortical sensory stream of sensation processing (see, for example, Grieve et al., 2000). These nuclei, however, are not known to have direct efferent relations with the primary motor region so as to promote its activation. Thus, other regions may be involved as intermediates between the posterior thalamus and primary motor regions.

#### Excitation of ventral thalamic nuclei can enhance responsiveness

Nuclei of the ventral thalamus (ventral lateral and ventral anterior) are known to have significant excitatory effects upon the motor regions, including the primary motor region (see, for example, Kurata, 2005). These thalamic nuclei seem to be under the significant control of subcortical circuits. In the context of the current model, we would like to emphasize, in this regard, the inhibitory control by the dorsal striatum (see, for example, DeLong and Wichmann, 2007). These ventral thalamic nuclei are not known to receive significant excitatory input from the posterior thalamic nuclei but from motor and related regions, likely including higher motor regions (see, for example, Rispal-Padel and Massion, 1970). But as we have seen earlier, it has been suggested that higher motor regions are active during maintenance, which by itself can enhance undesired responsiveness via the ventral thalamic nuclei. It is possible that activation of the relevant dorsal striatum by the same motor and related regions (see, for example, Jones et al., 1977) reduces, as noted, this thalamic activity during maintenance. However, the dorsal striatum is also not known to receive significant inhibitory input from the posterior thalamic nuclei and thereby to enable double inhibition or net excitation of the ventral thalamic nuclei when stimuli are sensed. Therefore, additional intermediate regions are required, as discussed below.

#### Control of dopamine release via the amygdala may be involved

One potentially relevant region on which the posterior thalamic nuclei are reported to exert significant excitatory effects is the central nucleus of the amygdala (see, for example, Pessoa and Adolphs, 2010). Among its other characteristics, this amygdalar region is reported to have significant excitatory effect on the release of dopamine from the substantia nigra pars compacta (see, for example, Gonzales and Chesselet, 1990), which, in turn, is believed to have a significant effect on the dorsal striatum. Dopamine release in the ventral striatum is believed to produce global excitatory effects, promoting the direct pathway of double inhibition, or net excitation of the relevant ventral thalamic nuclei (see, for example, DeLong and Wichmann, 2007].

To summarize, we suggest the plausibility of five intermediate regions between about the primary sensory region (including also the possibility of direct subcortical sensory activation of the pulvinar nuclei) and the primary motor region: primary sensory region → posterior thalamus → amygdala → substantia nigra pars compacta → dorsal striatum → ventral thalamus → motor cortex (including primary). Figure 4B presents this possible chain invoked by the stimulus. Note that Figure 4B also demonstrates the excitatory effects, discussed earlier, of the motor regions on both the relevant thalamic nuclei and the dorsal striatum. The relevant ventral thalamic nuclei reportedly show lasting burst activity evoked by their excitation (Steriade, 1997). Therefore, when their inhibition is suppressed, their effect on the relevant cortical regions may last more than a few tens of milliseconds, which was suggested above as being the elementary representation duration. Below we present EEG/ERP evidence that this activity lasts hundreds of milliseconds.

Theoretical chains like the one described above are somewhat fragile. For example, it appears that meticulous modeling of the dopamine effect in the context of the general function of the basal ganglia may result in changes in the preliminary model we presented. Another weakness in the proposed chain relates to the anatomical connections between the posterior thalamus and the central amygdala, and between the central amygdala and the substantia nigra pars compacta. Our knowledge regarding these connections stems mainly from several animal studies, and may be imprecise. Alternative chains may be possible, as well as potential shortcuts in the presented chain. Furthermore, as was stated previously for the hippocampus, the schema we described above hardly touches upon the possible functional features of the amygdala or of the basal ganglia.

Nevertheless, the requirement of a process for promoting responsiveness, which is likely to involve subcortical regions, is derivable directly from the processes described above as being necessary for modeling the go/no-go task. Furthermore, we cannot find many alternative regions and region chains that could underlie promotion of responsiveness aside from perhaps some shortcuts in the chain suggested above. Involvement of the basal ganglia, and in particular of its dopaminergic system, seems to be supported especially for strong, significant, or infrequent stimuli (see, for example, Schultz, 1998), and the same may be cited in support of the involvement of the amygdala (see, for example, LeDoux et al., 1990).

#### Enhanced responsiveness may depend upon stimulus frequency

Many of the protocols used for the go/no-go task employ imbalanced representation of the stimuli. Often the frequent stimulus is the one associated with the go response. Evidence exists for stronger activation of amygdalar representations by rare stimuli (see, for example, Halgren et al., 1980), which may be related to the general tendency of greater amygdalar responses to more significant stimuli, as mentioned above. The stronger activation in the amygdala may invoke stronger activation of the entire chain to promote responsiveness, as described above.

The stronger activation by the rare stimulus may simply express this greater sensitivity to it, which is a well-documented phenomenon in the neural tissues (see, for example, Eytan et al., 2003). Indeed, the greater response to a rare stimulus, or perhaps mainly the habituation of response to the frequent go stimulus, may be prominent from early sensation stages and manifest also at the amygdala, and therefore throughout the entire responsiveness chain, including the motor regions. If this is the case, promotion of responsiveness may be expected to be stronger for the stimulus associated with the no-go or avoid response. Note that if indeed, as suggested above, there is inhibition between similar representations at different loci, and thus maintenance activation of only one stimulus-response association is necessary, it may further affect habituation. For example, regular active maintenance of the more frequent go stimulus-response association can lead to further habituation. In such a case, the no-go-associated stimulus may elicit an even stronger response.

The go/no-go task and similar tests are utilized clinically in the diagnosis of ADHD. Below we suggest EEG/ERP evidence relating ADHD to deviation in activation of the responsiveness process discussed here. The problem may be anywhere in the pathway we presented in this section of primary sensory region → posterior thalamus → amygdala → substantia nigra pars compacta → dorsal striatum → ventral thalamus → motor cortex (including primary). The effect of methylphenidate, mediated through dopaminergic synapses, on the results of versions of go/no-go tests in ADHD, is also touched upon below in this context. Note that even if the pathophysiology can be related to other (non-dopaminergic) parts of the pathway, enhancement of relevant dopamine effect may still improve functionality.

To summarize this section, we found it necessary to model three processes that can possibly underlie the go/no-go task, and suggested a rough anatomic embodiment for their implementation. The neurophysiologic processes are: (i) memory maintenance, (ii) evoking perception by stimulus, and (iii) response enhancement (responsiveness).

## Manifestation of Neurophysiologic Processes in EEG

### Expression of the neurophysiologic processes in the EEG signal

Electroencephalography is a long standing technology with many aspects of practical use. Vast clinical and non-clinical research results seem to support the possibility of yet much wider practical use. This is further supported by the relative availability of this technology, in terms of usability and cost, which has been even further advanced over the last years. This is the motivation to search the manifestation of the modeled neurophysiologic processes in the EEG signal. We hope that sufficient specification of such manifestations will yield accurate and useful markers for the processes.

Not unlike other sampling technologies, the EEG signal has its limitations in representing the intricacy of neurophysiologic processes. It does not present the activity with a resolution of single units of representation (for example, cortical columns), it is less sensitive to activities in deep structures of the brain and its resolution of spatial localization is rather limited. Nevertheless, we suggest below a preliminary approximation of the manner in which neurophysiologic processes are manifested in the EEG signal.

#### The role of thalamocortical activation in the EEG signal

Various theories have been raised regarding the source of the EEG signal (see, for example, da Silva, 1991). An accepted theory relates much, if not all, of the signal, at least in standard frequency bands of analysis, to locally widespread EPSP activity, shared by many target neurons activated in synchrony. The afferent source of this spread synchrony is often considered to be a distant, often subcortical, nucleus. Thalamic nuclei are frequently mentioned in this context (see, for example, Olejniczak, 2006). There are nevertheless significant reports of other, non-thalamic nuclei that possess the afferent role in widespread cortical activity measured by EEG. Especially noteworthy are cortico-cortical activations described as significant (see, for example, Nunez and Srinivasan (2006)) which may manifest more in certain frequency bands. But even in these cases it is often possible to point at a thalamic nucleus whose activity bears a relation with the afferent source. For example, the anterior thalamus in the context of hippocampal activity and the pulvinar posterior thalamus in the context of higher perceptual cortical regions. Therefore, it may be possible to relate one or the other thalamic nuclei to most activities manifested in the EEG signal: each nucleus with its related widespread cortical activity, as far as position, duration, amplitude, and pattern are concerned, which may be approximated by dominant frequency bands. Again, some of these activities may be evoked by the relevant thalamic nuclei, whereas others may co-occur with activation in the thalamic nuclei. But it appears that there is only a limited set of candidate groups of thalamic nuclei, and therefore a limited set of possible neurophysiologic processes that could be manifest in the standard EEG signal. Although there may be some neurophysiologic processes manifested in the EEG signal without thalamic correlates, it is a reasonable initial approximation to expect the main bulk of the processes that are manifest in the signal to have such correlates.

#### Expected EEG manifestations of the go/no-go neurophysiologic processes

##### Memory maintenance: anterior nuclei

In the model we suggested that the anterior thalamic nuclei are related to memory processes as part of the hippocampal-thalamic-cortical pathways. Thus, it is possible to expect lasting EEG activity during the entire task as long as the association of stimuli with responses is maintained in memory. Based on the model, the spatial spread of this EEG activity is expected to involve relevant parietal, sensory, and motor regions.

##### Stimulus perception: pulvinar and surrounding nuclei

In the model we also suggested a role for posterior thalamic nuclei (specifically pulvinar nuclei) in perception. Thus, a relatively short activation, at least on the scale of tens of milliseconds (the suggested timescale of activation of a representation unit), may be expected over the relevant sensory and associated motor regions after stimulus presentation.

##### Responsiveness: ventral lateral and ventral anterior nuclei

In the model we suggested an important role of these ventral thalamic nuclei in the activation of relevant motor regions as part of the responsiveness process following the sensing of task-related stimuli. These nuclei appear to be heavily influenced by major subcortical motor-associated structures such as the cerebellum (see, for example, Kurata, 2005), and in the case of the go/no-go task, the basal ganglia (presented above). As was suggested for the go/no-go task, the dopaminergic effect on the basal ganglia may increase the strength and lengthen the duration of the cortical effect of these thalamic nuclei, and thereby enhance their EEG manifestation. It was also suggested above that rare stimuli may exert a stronger enhancing effect of this type.

In summary, the above basic EEG manifestations of the neurophysiologic processes that underlie the go/no-go task may be expected.

#### Other neurophysiologic processes expected to manifest in the EEG signal

Two other processes also appear to be active during the task and may therefore affect the EEG signal. The first is arousal, which relates to the intralaminar and midline thalamic nuclei (Van der Werf et al., 2002). This process is not task-related. The second process is that of sensation, relating to different thalamic nuclei that are specific to sensory modality. In the context of the go/no-go task, in which the stimuli evoke perception, which involves comparison with representations maintained in memory, it seems that the EEG activities related to sensation and to perception merge. This is due to their overlap in location, especially due to the limited spatial resolution of EEG, and in timing, because to the presumed small synaptic delay between primary and higher sensory regions in comparison with the thalamocortical effect on EEG.

#### Relation to events limits the sampled processes

We wish to draw attention to the distinction between the raw signal of a continuous EEG sample and the ERP signal after averaging the activity temporally related to the stimulus over a sufficient number of repetitions. Such averaging promotes the manifestation of processes that are time-locked to the stimulus, and averages out the manifestations of other on-going processes that are not time-locked. In the case of the go/no-go task we expect the memory maintenance process to be averaged out from the ERP signal and we therefore expect the ERP signal to consist of the superposition of the perception and responsiveness processes, each with its spatiotemporal manifestations, as described above. Below we evaluate the precision of these expectations by the current literature and with a comprehensive analysis of a relevant ERP dataset. Furthermore, we show how specific spatiotemporal differences in the ERP manifestations of the two processes enable the derivation of an effective and theory-based functional EEG/ERP marker.

### Matching the modeled processes with ERP literature of the go/no-go task

#### Major ERP patterns in response to go/no-go-related stimuli

The most robust finding of ERP analyses differentiating between go- and no-go-associated stimuli, regardless of the precise stimulation pattern, is the enhanced activity that occurs a few hundreds of milliseconds after the no-go-associated stimulus, most prominent over central and frontal regions (for example, Bokura et al., 2001). This activity is often marked as P3, a positive deflection with a peak that occurs some 300 ms or more after the onset of a task-relevant stimulus. This enhancement of the P3 activity for the no-go stimulus has been suggested to reflect response inhibition. But it has been shown that the P3 activity, together with related N2 activity (negative activity preceding P3 and peaking over 200 ms after stimulus onset), tend to increase in response to the go stimulus and to diminish in response to the no-go-associated stimulus among control subjects, when the go stimulus is presented less frequently (Nieuwenhuis and Yeung, 2003). Thus, it has been suggested that these N2 and P3 were associated with response preparation, or in our terms – responsiveness, enhanced by rare and conflicting stimuli rather than with response inhibition. Thus, overall, this activity is consistent in its reported spatiotemporal characteristics with our prediction regarding the expected the EEG/ERP manifestations of the responsiveness process.

Earlier ERP activity (e.g., the N1-P1 waves) seems to be evoked by both types of stimuli to a similar degree (Bokura et al., 2001). This shorter activity, on the scale of tens of milliseconds, seems to be localized according to the modality of stimulation, and it accords in spatiotemporal characteristics with our prediction regarding the EEG/ERP manifestations of the perception process. These two types of activity, the early one, which is similar to the go- and no-go-associated stimuli, and the late one, which designates the rarer no-go-associated stimulus, seem to comprise most if not all of the go/no-go ERP findings reported in the literature.

#### Findings in subjects with ADHD

There is compelling evidence of characteristic electroencephalographic (EEG) activity prevalent among subjects diagnosed with ADHD (for a review, see Loo and Barkley, 2005). The strength of this evidence is intriguing considering the fact that current diagnosis of ADHD is generally based on rather subjective behavioral measures. The main characteristic is a reduction in activity a few hundreds of milliseconds after the stimulus, as designated above (for example, Barry et al., 2003). The reduction of this activity (which we related above to the responsiveness process) among ADHD subjects is more pronounced with respect to the no-go-associated stimulus.

Whereas the literature regarding the P3 activity reports almost unanimously a reduction in ADHD subjects, reports regarding N2 activity differ. Some studies report that similarly to P3, N2 activity is also reduced in groups of ADHD subjects (for example, Wild-Wall et al., 2009; Smith et al., 2004). These results are consistent with the findings described in the studies presented above regarding the association between N2 and P3 activities in control subjects in response to no-go-associated rarer stimuli. But other studies report that N2 activity is enhanced in groups of ADHD subjects compared with controls (for example, Prox et al., 2007). A possible explanation for this apparent contradiction is that in studies that demonstrate enhanced N2 activity for ADHD subjects, the N2 peak appears earlier than in the studies showing similar reductions of N2 and P3 activities in ADHD. Some studies report enhanced early response in ADHD subjects up to 200 ms post-stimulus (for example, in N1 activity, Prox et al., 2007), compared with controls. Therefore, the contradictory findings regarding the N2 activity may represent some methodological differences regarding the temporal characterization of N2, and not necessarily different underlying neurophysiologic processes.

The enhanced earlier response in ADHD subjects is not necessarily presented in all the electrodes that demonstrate significant N1-P1 activity. Much of the spread N1-P1 activity, which was earlier related to the perception process, seems to be modality-specific in ADHD as well, and does not differ between go- and no-go-associated stimuli or from the activity demonstrated by control subjects. On the other hand, as demonstrated in another communication, this enhanced early ADHD activity is in-fact a deviant frontal-central responsiveness process (Shahaf et al., under review), which replaces the longer (N2-P3) activation that is manifest in control subjects and lacks in ADHD subjects.

The soundness of the ERP characteristics of ADHD is further demonstrated by its susceptibility to treatment, for example by methylphenidate. The above-mentioned ERP activities, particularly the P3 activity, often tend toward normalization following such treatment (for example, Sunohara et al., 1999; Verbaten et al., 1994). Because current opinion suggests that this treatment affects dopaminergic systems, plausibly in the basal ganglia, relevant ERP activity such as the P3 activity may reflect activation of cortical regions by subcortical nuclei, possibly involving the basal ganglia via relevant ventral thalamic nuclei. This seems to accord well with the anatomic embodiment suggested above for the pathway of the responsiveness process. Thus ADHD may be interpreted as some dysfunction along this pathway.

To summarize, we believe that the ERP signal of the go/no-go task can be decomposed into two elementary activity patterns, an early one that relates to the perception process presented above, and a longer one that relates to the responsiveness process presented above. We further believe that according to the literature, the responsiveness process is malfunctioning in ADHD.

### Matching the modeled processes with comprehensive ERP analysis

On top of the analysis of the literature, we developed a comprehensive data analysis method for the ERP signal, designed to identify any activity patterns, which characterize an experimental group of subjects. We employed this method on data sampled from 12 young adults suffering from ADHD and 12 age-matched control subjects. The subjects underwent an auditory go/no-go task. In this section we explain the principles of this method of comprehensive analysis and show the matching of the patterns it identifies with the neurophysiologic processes of perception and responsiveness, as they are presented above. Detailed discussion of the analysis method and results could be found elsewhere (Shahaf et al., under review). Detailed description of the experimental setup could be found (Fisher et al., 2011).

#### Principles of the comprehensive analysis

The data analysis consisted of two major steps: (i) comprehensive search for activity events consistent across subjects of the same experimental group and stimulus type; and (ii) analysis of spatiotemporal continuity between the events. Details on each step are presented below.

##### Search for consistent discrete ERP events (presented also in Shahaf et al., 2012)

**Figure 5:**
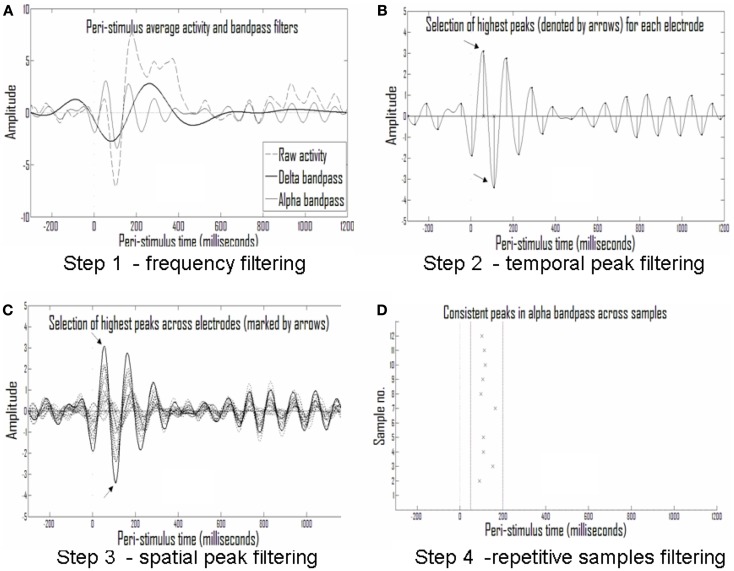
**Analysis of repetitive events**. **(A)** Averaged activity in each electrode and each sample is filtered into different frequency bands; shown are the delta (1.5–4 Hz) and alpha (7–13 Hz) frequency bands. **(B)** The largest half-waves in each frequency band are selected. **(C)** Only those that are also largest in activity compared with simultaneous activity in other electrodes are selected. **(D)** Only peaks that are repetitive across samples from the same experimental group (with temporal tolerance) are selected as repetitive events.

*First step: division into frequency bands*. As shown in Figure 5A, the first step in the analysis was to filter the raw ERP signal in each electrode into several frequency bands. The assumption was that an activity pattern consistent across subjects in one of the clinical groups and stimulation types will consistently manifest in one or more of the frequency bands. The frequency bands chosen partially overlapped, allowing redundancy in adjacent frequency bands but also reducing misses of consistent activity patterns in somewhat different frequencies in different subjects.

*Second step: discretization to events of peak time and amplitude*. The eight frequency bands selected (0.5–1.25, 1–2, 1.5–4, 3–8, 7–13, 12–18, 17–23, and 22–30 Hz) were sufficiently narrow to produce near symmetrical half-waves (Figure 5A). Selection of these frequency bands enabled discretization of each half-wave and its description by its peak amplitude and timing. This data reduction to practically symmetric half-waves allowed approximation of the entire envelope of the half-wave by the discrete values without significant loss of information (Figure 5B).

*Third step: narrowing to the strongest events in time and space*. For the sake of initial pattern analysis, to find the strongest consistent activities characterizing the response in a given clinical group to a given stimulus type, the largest half-wave peaks for each electrode, and each frequency band were selected. In Figure 5B the times of the largest peaks for each electrode are denoted by *x* on the abscissa. In addition to selecting only the strongest activities per electrode and frequency band, we set another threshold on the rank of this peak among the activities present in the other electrodes at the same time, selecting only activities that were both strongest in the given electrode over time and across electrodes in space. Figure 5C depicts this second spatial threshold.

*Fourth step: looking for consistent events across subjects*. Thus, a raster plot of threshold-crossing events was formed with limited loss of information about the repetitive patterns across the ERP of each clinical group in response to a given stimulus. A moving window was then applied on event timing relative to stimulus in order to identify repetitive activities across subjects (Figure 5D).

##### Assessment of spatiotemporal continuity of events

**Figure 6:**
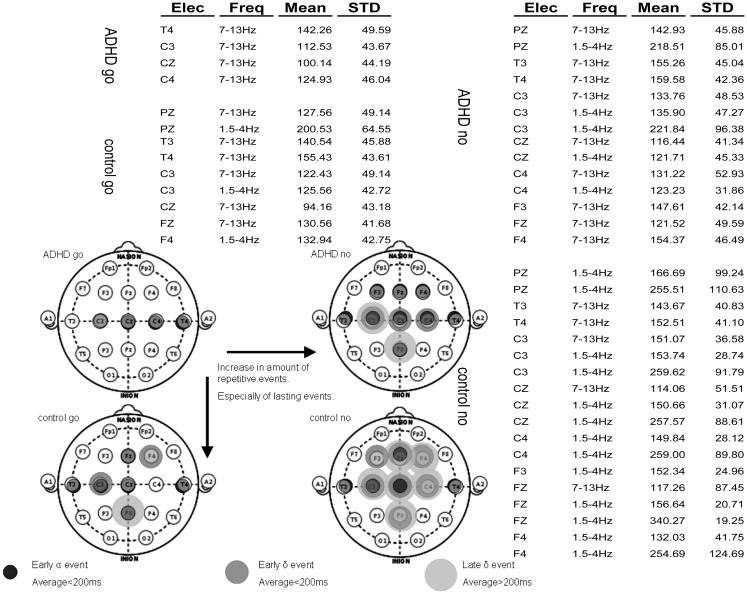
**Summary of repetitive events in the various experimental conditions: ADHD-go (top right), ADHD-no-go (top left), control-go (bottom right), control-no-go (bottom left)**. Three types of repetitive activity can be found by applying the analysis method described: (i) early alpha event with average peak time (across samples) below 200 ms, (ii) early delta event with average peak time below 200 ms, and (iii) late delta event with average peak time above 200 ms. The raw data of average event time across subjects and standard deviation are presented in the background table.

After applying the algorithm for analysis of consistent ERP events, the vast majority of activities characterizing each group were found in the 1.5–4 and 7–13 Hz frequency bands. The rest of the analysis focuses on these bands. Figure 6A presents a table of all consistent events found in the four experimental conditions: ADHD-go, control-go, ADHD-no-go, and control-no-go in the frequency bands 1.5–4 Hz (∼delta) and 7–13 Hz ( ∼alpha). For each event, the average amplitude time across subjects and its standard deviation are presented.

Note that more events were found in the control than in the ADHD condition. In addition, more events were found in the no-go than in the go condition. Moreover, early events (average<200 ms) can also be seen in all experimental conditions in some central (C), some temporal (T), and often some frontal (F) electrodes in the alpha frequency band. In some experimental conditions such early alpha events are also found at Pz. Overall, both early (average<200 ms) and late (average>200 ms) delta events are more prevalent in the control than in the ADHD condition and in the no-go than in the go condition. Figure 6B summarizes topographically the consistent events found in each experimental condition based on the distinction between alpha and delta events and further between early (average<200 ms) and late (average>200 ms) delta events.

**Figure 7:**
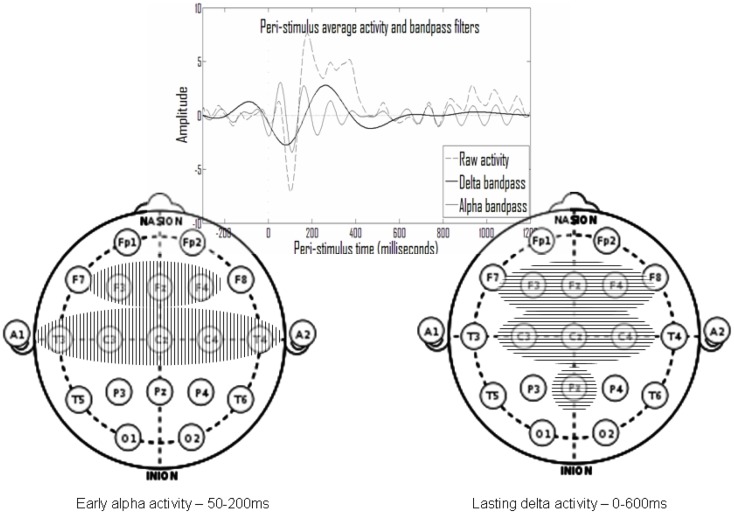
**In the top inset note that activity peaks in the delta and in the alpha ranges appear to be part of larger continuous activity**. Summarizing the results presented in Figure 6, there is repetitive early alpha activity in at-least one experimental condition in eight electrodes (left) and repetitive prolonged delta activity in at-least one experimental condition in seven electrodes (right). Six of these electrodes overlap and there is also partial temporal overlap.

Figure 6 shows that electrodes with late delta events also tend to have early delta events. The inset in Figure 7 presents a typical average-ERP response in the same electrode of a control subject in the go condition, with the filtered activities in the delta and alpha frequency bands. Note that the early and late delta activity peaks are part of two half-waves in a continuous activity of several 100 ms following the stimulus. Similarly, in the alpha frequency band the peaks were part of a continuous activity of a shorter duration. Such temporal continuity is consistent across electrodes, subjects, and experimental conditions. Therefore, early (average<200 ms) and late (average>200 ms) delta activities in certain electrodes represent a single prolonged activity in these electrodes, often until approximately 600 ms after stimulus onset.

Overall, the activities found belong either to an early set, in the alpha frequency band at 50–200 ms post-stimulus, or to a prolonged set in the delta frequency band beginning around stimulus onset until 600 ms post-stimulus. Such a summary of two sets is presented in Figure 7, which includes the electrodes of each set. Note that there is significant overlap in time and space (participating electrodes) between the two activity sets.

#### Interpretation of results

The analysis results suggest that the go/no-go task ERP activity emerges from two processes spread over time and space. The first process, which is termed *early*, seems to manifest strongly at 50–200 ms following the stimulus in the alpha frequency band, mainly at the temporal and centrofrontal electrodes. The second process, which is termed *prolonged*, seems to manifest strongly at 0–600 ms following the stimulus in the delta frequency band, mainly at centrofrontal and parietal electrodes. There is a clearly notable spatial overlap between the two suggested activities in the time range of 50–200 ms at the centrofrontal electrodes. The early activity appears in both controls and ADHD subjects and for each group, both in the go and in the no-go conditions. The prolonged activity, however, is most evident in the control-no-go condition, to a lesser degree in the ADHD-no-go condition and in the control-go condition, and least in the ADHD-go condition.

In spatiotemporal terms these activities are consistent with the predicted manifestations of the perception (early) and responsiveness (prolonged) processes, as they are modeled in the first section. The responsiveness-related prolonged activity seems to overlap spatiotemporally with the P3 and late N2 activities, characteristic in the literature of the control group and of the no-go condition. The activity related to the enhanced early responsiveness, characteristic of the ADHD group (Figure 6 – ADHD-no-go condition, F electrodes), seems to overlap the enhancement of earlier deflections, also reported in the literature. As predicted in the first section, this responsiveness process is enhanced by rare no-go stimuli.

Because of the “corrective” effect of methylphenidate, reported in the literature, producing in ADHD subjects a more control-like pattern, the responsiveness process may involve cortical disinhibition by basal ganglia, mediated by ventral thalamic nuclei. This involvement is consistent with the pathway suggested in the first section.

The distributed early activity involving the temporal electrodes, which is manifested similarly in all experimental conditions, is spatiotemporally consistent with the expected manifestations of the perceptual process, and it also accords with the conventional interpretation in the literature of early ERP deflections as representing perceptual processes. The distribution of this consistent early activity over temporal (plausibly auditory) and sensorimotor regions seems to be in accordance with this interpretation.

Overall, we demonstrated in this section the matching between a comprehensive bottom-up data analysis approach, as well as the relevant up-to-date literature, with the predictions of the theoretical model of underlying neurophysiologic processes and their manifestation in EEG.

### Deriving an EEG marker for ADHD by segregating process manifestations

As was stated above there are spatiotemporal differences between the EEG manifestations of the perception and responsiveness processes, However there is also significant overlap, both theoretically driven and experimentally demonstrated. This overlap may have significant impact upon the precision of most ERP analysis methods – for example of standard ERP wave amplitudes and latencies. Instead we suggest emphasizing the differences in the manifestations of the processes.

**Figure 8:**
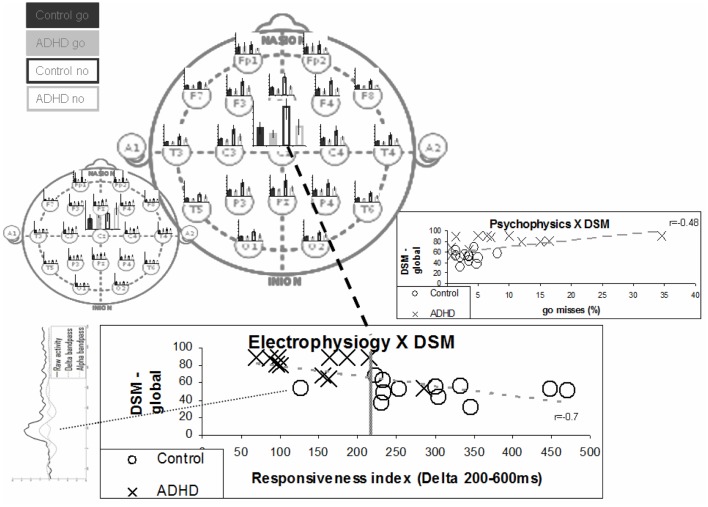
**“Responsiveness index” – a single electrode biomarker**. The bottom chart presents the distribution of the various subjects in terms of ERP responsiveness index and their DSM global grade. The responsiveness index actually denotes the late activity in the no-go condition (200–600 ms post-stimulus) at the Cz electrode. The average late activities for the various electrodes are represented in the top main left topographic image to clarify it distributed nature. The early activities are represented in the small topographic image to emphasize the differences between the two distributions. Note that the separation between groups is better than the one obtained for the best psychophysical index – presented in the inset on the right (also correlation with DSM is stronger). Note that one ADHD subject actually shows both electrophysiological and DSM characteristics more similar to control subjects. Note that the one deviant subject has ERP activity which does not involve clear N1, N2, and P2 activities (bottom left inset).

Even simple temporal segregation seems to suffice. In Figure 8, we show the effect of simple integration of the delta activity in the period of 200–600 ms as a marker for the responsiveness process, which excludes early effect of the perception process. The bottom chart presents a comparison of this activity with the DSM questionnaire (Conners) evaluation results. Note that the one ADHD subject who had low Conners scores also had strong late activity (both similar to controls). The inset on the right shows a group comparison with the percentage of go misses, the psychophysical index which best differentiated ADHD and control subjects. Note that this best psychophysical index is significantly less effective in the distinction between the ADHD and control subjects, when compared to the electrophysiological biomarker. Note the left bottom inset presenting the data of the deviant subject (marked in yellow). It resembles prefrontal activity in other subjects (not shown) and may represent misplacement of the Cz electrode (see also related prefrontal findings in Alexander et al., 2008).

Thus it is possible to derive a theory-based simple markers for the functionality of the neurophysiologic processes underlying behavioral function vs. dysfunction, as is demonstrated for ADHD. This is done by harnessing precisely of elementary differences in the spatiotemporal manifestations of the processes.

## General Conclusion Regarding Neurophysiologic Modeling and EEG Manifestations

### A limited set of neurophysiologic processes manifesting in EEG

In the present study we demonstrated the possibility that a rather limited number of neurophysiologic processes can underlie the EEG signal. To demonstrate this possibility, we employed a top-down model-derived analysis, based on the assumption that the neurophysiologic processes manifest in EEG relate to a set of thalamic nuclei. We further matched the modeled processes with a bottom-up analysis of the literature as well as with a comprehensive data analysis method, and showed the precision, which could be derived from it. In the context of the go/no-go task, we discussed five types of neurophysiologic processes that seem to have such an associated thalamic center: memory maintenance, perception, responsiveness, arousal, and sensation. We are aware of only one additional relevant thalamic center and thereby neurophysiologic process, which could manifest in EEG – the executive function, which seems to be related to the dorsal medial thalamic nuclei (see, for example, Wells, 1966).

We believe the EEG signal to be a superposition of the manifestation of this limited number of underlying basic neurophysiologic processes, which are currently thought to be six. In support of this possibility, at least as far as ERP is concerned, consider the limited set of typical wave deflections shared by various experimental tasks; these manifestations of underlying neurophysiologic processes show a significant spatiotemporal overlap. As we demonstrated we believe that it is usually possible to segregate these manifestations quite precisely, with rather simple markers, on the basis of differing spatiotemporal characteristics, but this must be proven one dataset at a time.

### A limited set of types of neurophysiologic processes in general

In the first section of this work we provided a thorough specification of the neurophysiologic processes underlying the behavioral task of go/no-go, which is often employed for the evaluation of attention. Similar modeling and specification of underlying neurophysiologic processes can be suggested for other aspects of behavior, as long as it can be defined operationally in a like manner. On this basis, it may be possible to relate behavioral dysfunction to problems in one or more of the underlying neurophysiologic processes and to describe any behavioral effects a treatment may have as stemming from its measured effects on these processes.

We specified in some detail three neurophysiologic processes (memory maintenance, perception, and responsiveness), touched upon another two in the context of EEG manifestation (arousal and sensation), and mentioned an additional one (executive function). Each of these processes can be detailed, for example, by specifying different sensory modality involved in one task or another. Furthermore, it is clear that some additional processes are involved in various behavioral functions. For example, it seems necessary to model the processes involving activity-dependent change, which are relevant to exploration and learning behavior. But it is not unrealistic to conceive of a rather limited and comprehensive set of such neurophysiologic processes that underlie any behavior as long as it is operationally measurable. It is likely that most of these processes are among those measurable with EEG (or with other techniques not discussed in our work), as the interaction between cortex and thalamic nuclei occurs in most significant neurophysiologic processes. Future work should demonstrate the applicability of the above approach to other types of operationally defined behavioral function and dysfunction.

Operational definitions exist for much of behavioral function and dysfunction, though there are often significant variations in the definitions offered for the same behavior. We hope and believe it will be possible to derive from such a limited core of underlying neurophysiologic processes a clear-cut definition of the spectrum of possible behavioral functions and dysfunctions in a comprehensive manner. This may enable comprehensive assessment, unification, and sharpening of current operational definitions. The possibility that these core neurophysiologic processes, which underlie behavior, are measurable directly with the available technology of EEG, via simple and precise markers, may have major practical significance, both clinical and non-clinical.

### Impact on the research of neuronal representation

Finally we discuss the impact the approach we present may have on the research of neuronal representation. The characteristics upon which we based our model are elementary. The evaluation of the impact of more fine-tuned may be a neuropsychological eye-opener and we call for such a systematic effort. Undoubtedly throughout the modeling and further on in the relation of the modeled processes with the EEG signal, we employed sometimes less sound assumptions regarding neuronal representation. Evaluation and correction of the characteristics seems to us of major significance.

## Conflict of Interest Statement

The authors declare that the research was conducted in the absence of any commercial or financial relationships that could be construed as a potential conflict of interest.
